# Supplementary material for: MicroRNAs promote skeletal muscle differentiation of mesodermal iPSC-derived progenitors
Source: Nat Commun. 2017 Nov 1;8:1249. doi: 10.1038/s41467-017-01359-w (PMC5665910; doi:10.1038/s41467-017-01359-w)
Supplement: Supplementary file 1 — Supplementary Information [file 41467_2017_1359_MOESM1_ESM.pdf]

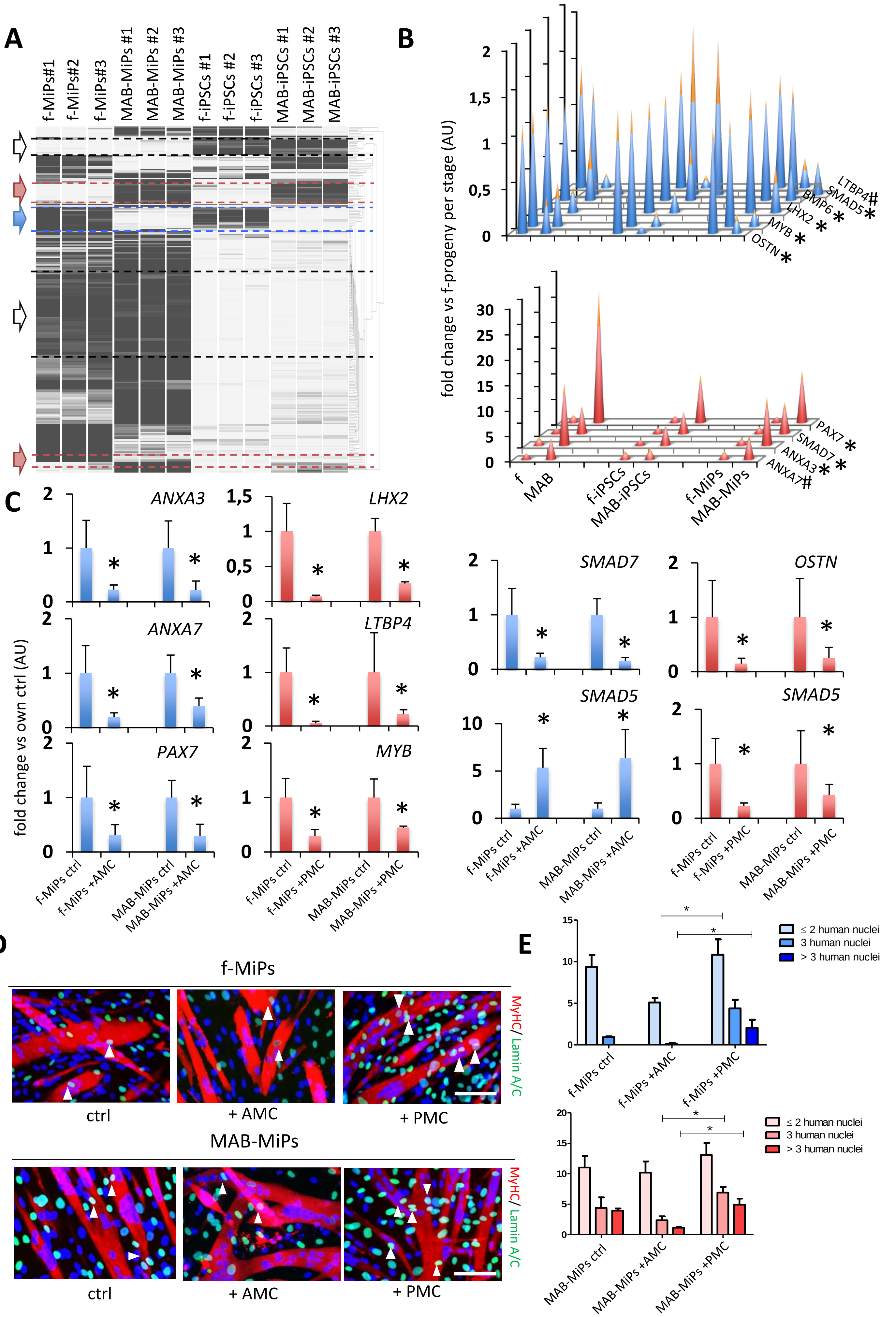
A
f-MiPs#1
f-MiPs#2
f-MiPs#3
MAB-MiPs #1
MAB-MiPs #2
MAB-MiPs #3
f-iPSCs #1
f-iPSCs #2
f-iPSCs #3
MAB-iPSCs #1
MAB-iPSCs #2
MAB-iPSCs #3
B
fold change vs f-progeny per stage (AU)
LTBP4#
SMAD5*
BMP6*
LHX2*
MYB*
OSTN*
PAX7*
SMAD7*
ANXA3*
ANXA7#
f
MAB
f-iPSCs
MAB-iPSCs
f-MiPs
MAB-MiPs
C
fold change vs own ctrl (AU)
ANXA3
ANXA7
PAX7
LHX2
LTBP4
MYB
SMAD7
SMAD5
OSTN
SMAD5
f-MiPs ctrl
f-MiPs +AMC
MAB-MiPs ctrl
MAB-MiPs +AMC
f-MiPs +PMC
MAB-MiPs +PMC
D
f-MiPs
MAB-MiPs
ctrl
+ AMC
+ PMC
MyHC/ Lamin A/C
E
≤ 2 human nuclei
3 human nuclei
> 3 human nuclei
f-MiPs ctrl
f-MiPs +AMC
f-MiPs +PMC
MAB-MiPs ctrl
MAB-MiPs +AMC
MAB-MiPs +PMC

Supplementary Figure 1. Gene shortlist for propensity perturbation in MiPs. (**A**) Heatmap (z-test) of DE genes (threshold, >10 norm counts) discriminating fibroblast- and MAB-MiPs, and fibroblast- and MAB-iPSCs. Emerging patterns of stage-associated (white arrow), fibroblast-progeny-associated (blue arrow) and MAB-progeny-associated (red arrows) are highlighted. (**B**) Histograms depicting qPCR-assessed expression levels of shortlisted genes, comparing MAB- vs fibroblast-derived cells across stages (somatic, iPSCs, MiPs). Blue bars depict average values of genes enriched in fibroblast-MiPs, red bars relate to genes enriched in MAB-MiPs, yellow spikes depict st.dev values. *, $P<0.05$ MAB- vs fibroblast- at all stages; #, $P<0.05$ at somatic and MiP, but not at iPSC stage; Kruskal Wallis and Mann-Whitney U test; n=3replicates/clone. (**C**) Expression levels in MiPs and AMC- or PMC-treated MiPs (gene targeting cocktails) of targeted genes. *, $P<0.05$ vs own ctrl (scramble), Kruskal Wallis and Mann-Whitney U test; n=3replicates/clone. **(D-E)** Quantification of MiP myogenic propensity in co-culture with C2C12 myoblasts after seven days of differentiation. Myotubes with three or more nuclei were counted as well as human nuclei contributing to chimeric myotubes. Representative fields and quantitation of chimeric myotubes are presented. $P<0.05$ vs treated and non treated. Kruskal Wallis and Mann-Whitney U test; n=3replicates/clone. f-, fibroblast-derived; AMC, anti-myogenic cocktail (gene-based), PMC, pro-myogenic cocktail (gene-based), scale bar approximately 100µm.

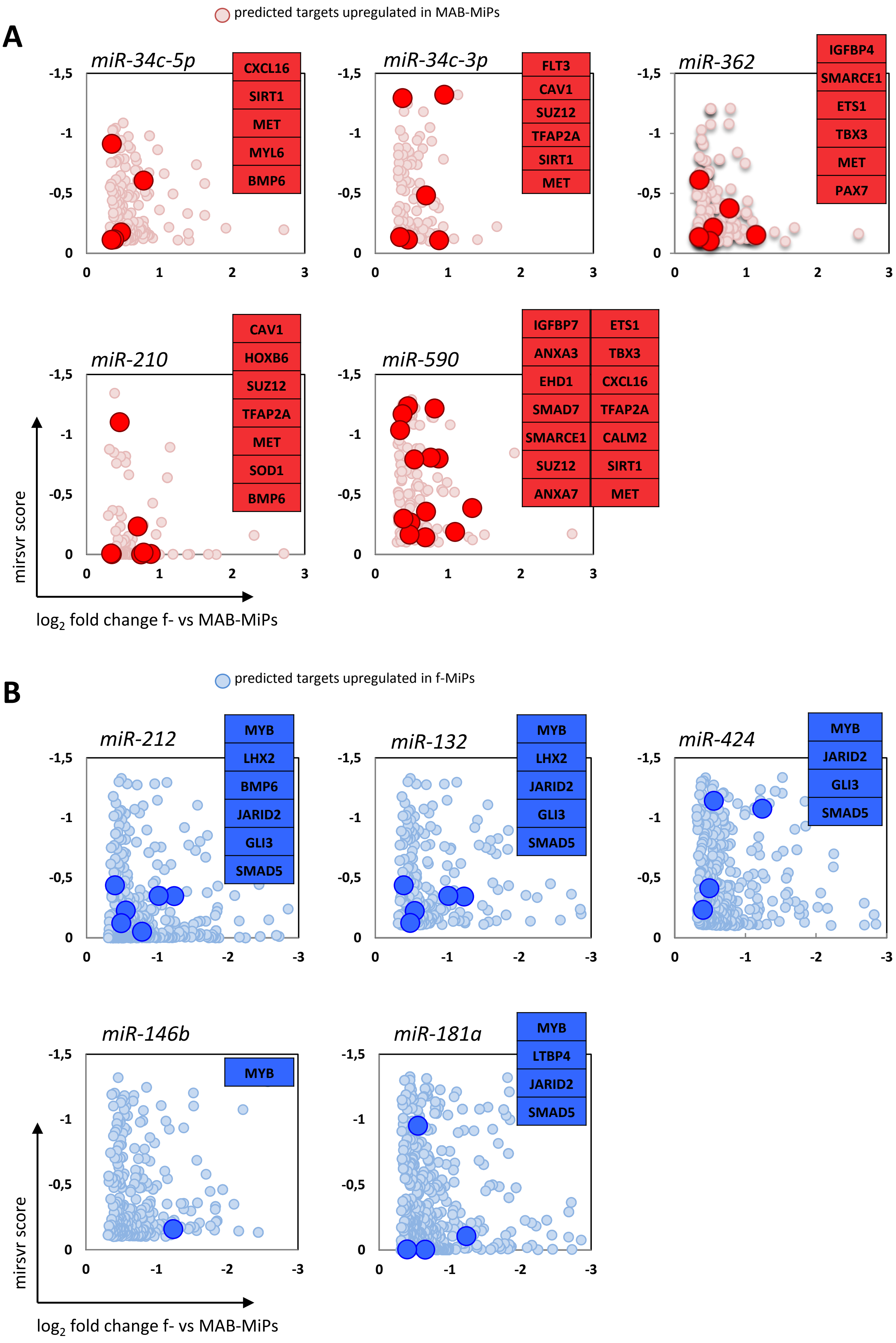
A
predicted targets upregulated in MAB-MiPs
miR-34c-5p
CXCL16
SIRT1
MET
MYL6
BMP6
miR-34c-3p
FLT3
CAV1
SUZ12
TFAP2A
SIRT1
MET
miR-362
IGFBP4
SMARCE1
ETS1
TBX3
MET
PAX7
miR-210
CAV1
HOXB6
SUZ12
TFAP2A
MET
SOD1
BMP6
miR-590
IGFBP7
ANXA3
EHD1
SMAD7
SMARCE1
SUZ12
ANXA7
ETS1
TBX3
CXCL16
TFAP2A
CALM2
SIRT1
MET
-1,5
-1
-0,5
0
0
1
2
3
mirsvr score
log2 fold change f- vs MAB-MiPs
B
predicted targets upregulated in f-MiPs
miR-212
MYB
LHX2
BMP6
JARID2
GLI3
SMAD5
miR-132
MYB
LHX2
JARID2
GLI3
SMAD5
miR-424
MYB
JARID2
GLI3
SMAD5
miR-146b
MYB
miR-181a
MYB
LTBP4
JARID2
SMAD5
-1,5
-1
-0,5
0
0
-1
-2
-3
mirsvr score
log2 fold change f- vs MAB-MiPs

Supplementary Figure 2. Filtering the candidates from miRNA-seq comparison by means of RNA-seq-based indications. The mirsvr score vs fold change plots in this Figure illustrate the predicted targets of miRNA-seq-based candidates among RNA-seq-based interesting hits. Specifically, charts in (**A**) depict the predicted targets (interesting hits are highlighted) of fibroblast-MiP-enriched miRNAs among genes upregulated in MAB-MiPs. Conversely, charts in (**B**) depict the predicted targets (interesting hits are highlighted) of MAB-MiP-enriched miRNAs among genes upregulated in fibroblast-MiPs. f-, fibroblast-derived.

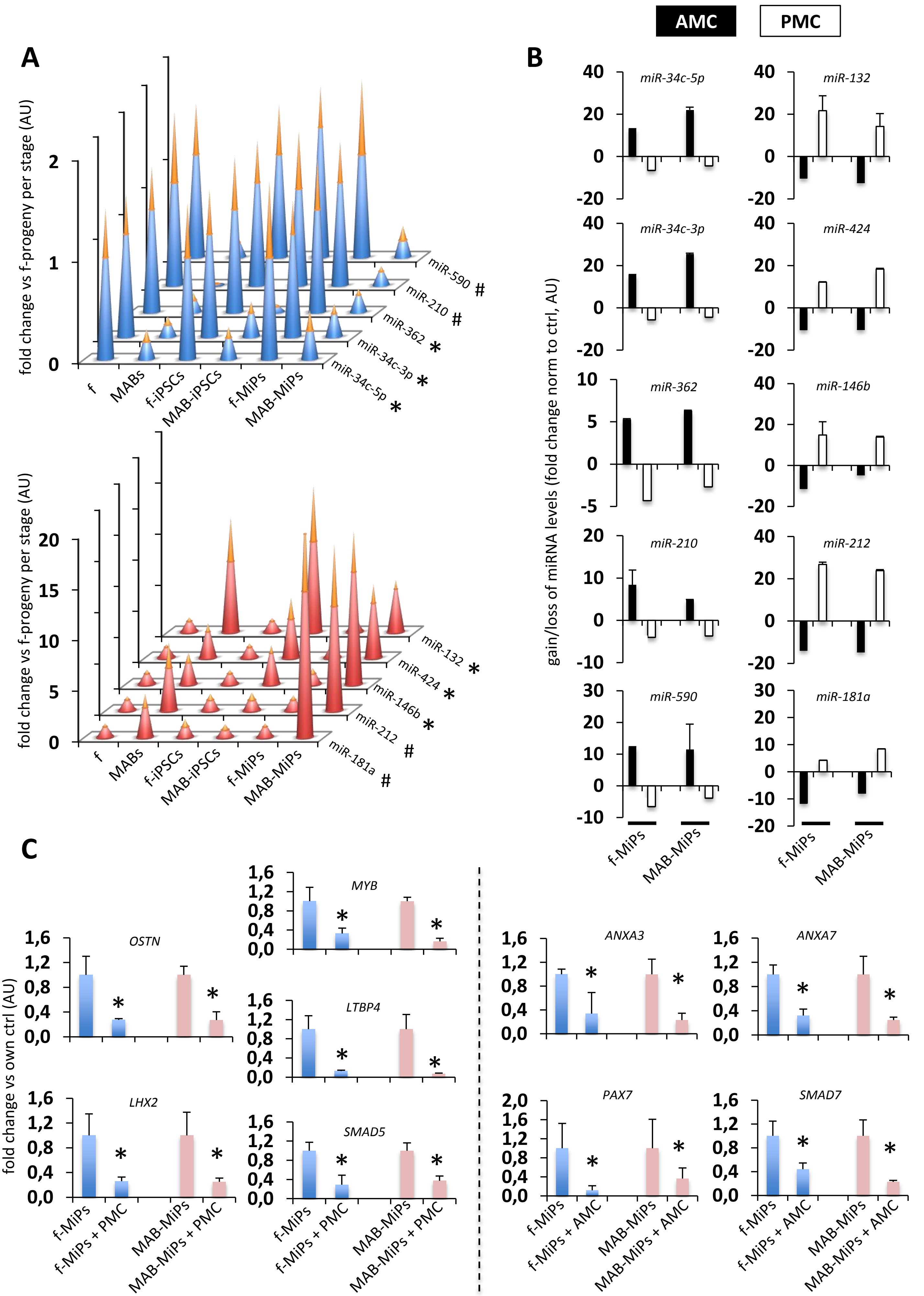
AMC
PMC
A
fold change vs f-progeny per stage (AU)
f
MABs
f-iPSCs
MAB-iPSCs
f-MiPs
MAB-MiPs
miR-590 #
miR-210 #
miR-362 *
miR-34c-3p *
miR-34c-5p *
miR-132 *
miR-424 *
miR-146b *
miR-212 #
miR-181a #
B
gain/loss of miRNA levels (fold change norm to ctrl, AU)
miR-34c-5p
miR-132
miR-34c-3p
miR-424
miR-362
miR-146b
miR-210
miR-212
miR-590
miR-181a
C
fold change vs own ctrl (AU)
OSTN
MYB
LTBP4
LHX2
SMAD5
ANXA3
ANXA7
PAX7
SMAD7
f-MiPs + PMC
MAB-MiPs + PMC
f-MiPs + AMC
MAB-MiPs + AMC

Supplementary Figure 3. Perturbing miRNAs and predicted targets with AMC and PMC containing miR-mimics and anti-miRs. (**A**) qPCR-based validation miRNA-seq-based hits across stages. Blue bars depict average values of genes enriched in fibroblast-MiPs, red bars relate to genes enriched in MAB-MiPs, yellow spikes depict st.dev values. *, $P<0.05$ MAB- vs fibroblast- at all stages; #, $P<0.05$ at somatic and MiP, but not at iPSC stage; Kruskal Wallis and Mann-Whitney U test; n=3replicates/clone. (**B**) qPCR-based evaluation of target miRNA levels after AMC or PMC administration to MiPs *in vitro*. N=3 replicates/clone; depicted are average±st.dev bars. **(C)** qPCR-based assay of expression levels of predicted targets of perturbed miRNAs in presence of AMC or PMC. *, $P<0.05$ vs own ctrl (scramble), Kruskal Wallis and Mann-Whitney U test; n=3replicates/clone. f-, fibroblast-derived; AMC, anti-myogenic cocktail (miRNA-based), PMC, pro-myogenic cocktail (miRNA-based).

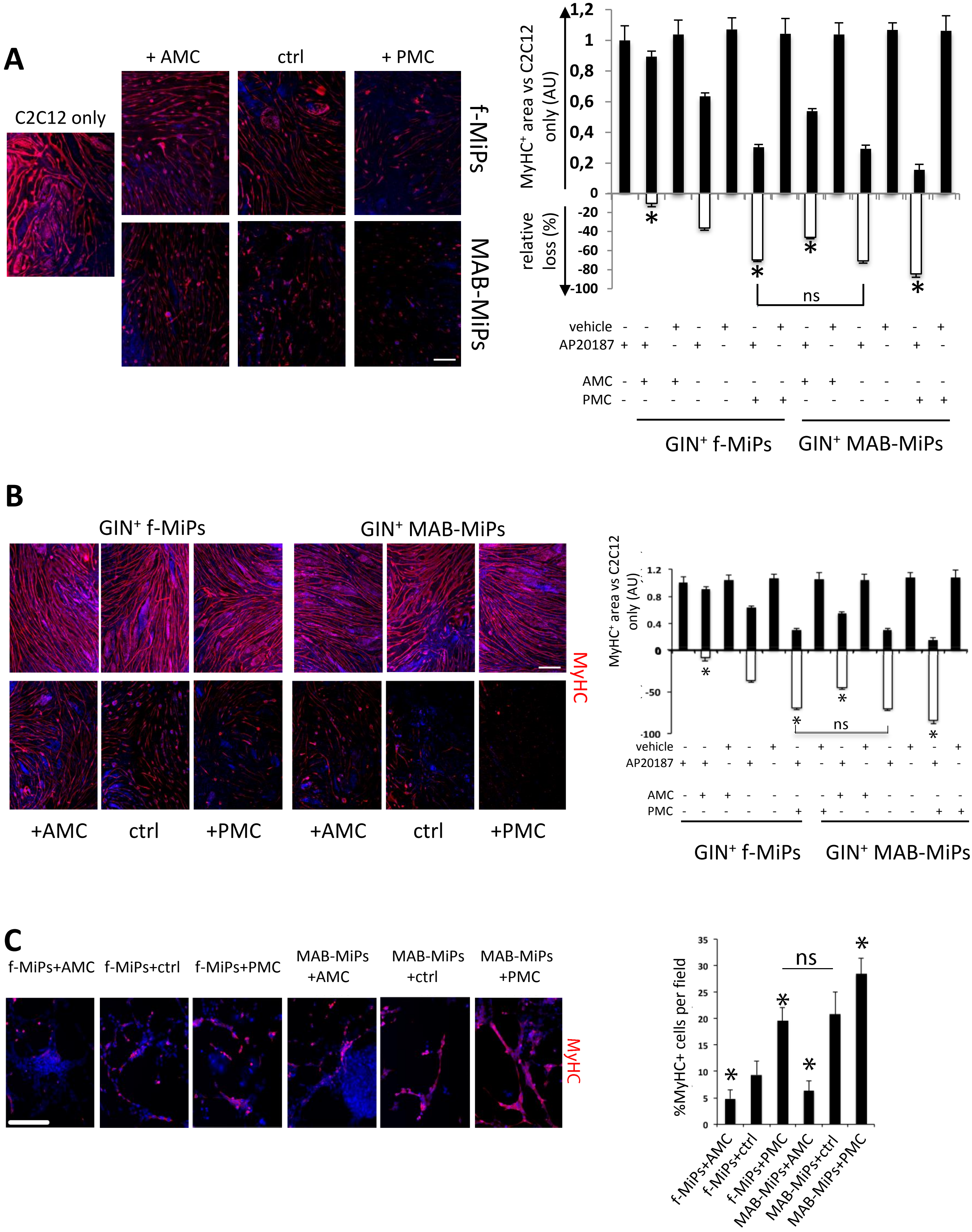
A
C2C12 only
+ AMC
ctrl
+ PMC
f-MiPs
MAB-MiPs
MyHC+ area vs C2C12 only (AU)
relative loss (%)
1,2
1
0,8
0,6
0,4
0,2
0
-20
-40
-60
-80
-100
ns
vehicle
AP20187
AMC
PMC
GIN+ f-MiPs
GIN+ MAB-MiPs
B
GIN+ f-MiPs
GIN+ MAB-MiPs
+ vehicle
+ AP20187
+AMC
ctrl
+PMC
+AMC
ctrl
+PMC
MyHC
MyHC+ area vs C2C12 only (AU)
1.2
0.8
0.4
0
-50
-100
ns
vehicle
AP20187
AMC
PMC
GIN+ f-MiPs
GIN+ MAB-MiPs
C
f-MiPs+AMC
f-MiPs+ctrl
f-MiPs+PMC
MAB-MiPs +AMC
MAB-MiPs +ctrl
MAB-MiPs +PMC
MyHC
%MyHC+ cells per field
35
30
25
20
15
10
5
0
ns
f-MiPs+AMC
f-MiPs+ctrl
f-MiPs+PMC
MAB-MiPs+AMC
MAB-MiPs+ctrl
MAB-MiPs+PMC

Supplementary Figure 4. Co-culture analysis performed in presence of AP20187.

(A) Reverse quantitation of MiP myogenic propensity in co-culture with C2C12 myoblasts after seven days of differentiation. AP20187 induces death of MiP-chimeric myotubes, therefore high propensity associates with high mortality (as depicted in the scheme on the left). Representative fields and quantitation of MyHC signal are presented. ns, non-significant difference; *, $P<0.05$ vs own ctrl (scramble), Kruskal Wallis and Mann-Whitney U test; n=3replicates/clone. f-, fibroblast-derived; AMC, anti-myogenic cocktail (gene-based), PMC, pro-myogenic cocktail (gene-based), scale bar approximately 100µm (B) Reverse quantitation of MiP myogenic propensity in co-culture with C2C12 myoblasts after seven days of differentiation. AP20187 induces death of MiP-chimeric myotubes, therefore high propensity associates with high mortality (as depicted in the scheme on the left). Representative fields and quantitation of MyHC signal are presented, scale bar approximately 100µm (**C**) Application of AMC and PMC mixes to induced MiP differentiation toward skeletal muscle lineage *in vitro*. Quantitation of differentiation efficiency is reported on the right. f-, fibroblast-derived; AMC, anti-myogenic cocktail , PMC, pro-myogenic cocktail, scale bar approximately 100µm.

Supplementary Table 1. List for esiRNA target sequences used

Target gene	cDNA Target sequence
PAX7	CCAAGATTCTTTGCCGCTACCAGGAGACCGGGTCCATCCGGCCTGGG<br>GCCATCGGCGGCAGCAAGCCCAGACAGGTGGCGACTCCGGATGTAG<br>AGAAAAAGATTGAGGAGTACAAGAGGGAAAACCCAGGCATGTTCAGCT<br>GGGAGATCCGGGACAGGCTGCTGAAGGATGGGCACTGTGACCGAAG<br>CACTGTGCCCTCAGGTTTAGTGAGTTCGATTAGCCGCGTGCTCAGAAT<br>CAAGTTCGGGAAGAAAGAGGAGGAGGATGAAGCGGACAAGAAGGAG<br>GACGACGGCGAAAAGAAGGCCAAACACAGCATCGACGGCATCCTGGG<br>CGACAAAGGGAACCGGCTGGACGAGGGCTCGGATGTGGAGTCGGAA<br>CCTGACCTCCCACTGAAGCGCAAGCAGCGACGCAGTCGGACCACATT<br>CACGGCCGAGCAGCTGGAGGAGCTGGAGAAGGCCTTTGAGAGGACC<br>CACTACCCAGACATATACACCCGCGAGG
SMAD5	GGACCAGGAAGTCCATTTCAGCTCCCAGCTGATACGCCTCCTCCTGCC<br>TATATGCCACCTGATGATCAGATGGGTCAAGATAATTCCCAGCCTATG<br>GATACAAGCAATAATATGATTCCTCAGATTATGCCCAGTATATCCAGCA<br>GGGATGTTCAGCCTGTTGCCTATGAAGAGCCTAAACATTGGTGTTCAA<br>TAGTCTACTATGAATTAAACAATCGTGTTGGAGAAGCTTTTCATGCATC<br>TTCTACTAGTGTGTTAGTAGATGGATTCACAGATCCTTCAAATAACAAA<br>AGTAGATTCTGCTTGGGTTTGTTGTCAAATGTTAATCGTAATTCGACAA<br>TTGAAAACACTAGGCGACATATTGGAAAAGGTGTTCATCTGTACTATGT<br>TGGTGGAGAGGTGTATGCGGAATGCCTCAGTGACAGCAG
OSTN	CACATTTCATCCTGGCTGTGACACTGACACTGTGGAGCTCAGGAAAAG<br>TCCTCTCAGTAGATGTAACAACAACAGAGGCCTTTGATTCTGGAGTCAT<br>AGATGTGCAGTCAACACCCACAGTCAGGGAAGAGAAATCAGCCACTGA<br>CCTGACAGCAAAACTCTTGCTTCTTGATGAATTGGTGTCCCTAGAAAAT<br>GATGTGATTGAGACAAAGAAGAAAAGGAGTTTCTCTGGTTTTGGGTCT<br>CCCCTTGACAGACTCTCAGCTGGCTCTGTAGATCACAAAGGTAAACAG<br>AGGAAAGTAGTAGATCATCCAAAAAGGCGATTTGGTATCCCCATGGAT<br>CGGATTGGTAGAAACCGGCTT
MYB	GGGCAGTAGAGCTTGGACAGAAAGAAAAGAAACTTGGTGTTAGGTAAT<br>TGACTATGCACTAGTATTTCAGACTTTTTAATTTTATATATATATACATTT<br>TTTTTCCTTCTGCAATACATTTGAAAACTTGTTTGGGAGACTCTGCATTTT<br>TTATTGTGGTTTTTTTGTTATTGTTGGTTTATACAAGCATGCGTTGCACTT

	CTTTTTTGGGAGATGTGTGTTGTTGATGTTCTATGTTTTGTTTTGAGTGT<br>AGCCTGACTGTTTTATAATTTGGGAGTTCTGCATTTGATCCGCATCCCCT<br>GTGGTTTCTAAGTGTATGGTCTCAGAACTGTTGCATGGATCCTGTGTT
LXH2	GCTCGGGACTTGGTTTATCACCTCAACTGCTTCACGTGCACCACGTGT<br>AACAAGATGCTGACCACGGGCGACCACTTCGGCATGAAGGACAGCCT<br>GGTCTACTGCCGCTTGCACTTCGAGGCGCTGCTGCAGGGCGAGTACC<br>CCGCACACTTCAACCATGCCGACGTGGCAGCGGCGGCCGCTGCAGC<br>CGCGGCGGCCAAGAGCGCGGGGCTGGGCGCAGCAGGGGCCAACCC<br>TCTGGGTCTTCCCTACTACAATGGCGTGGGCACTGTGCAGAAGGGGC<br>GGCCGAGGAAACGTAAGAGCCCGGGCCCCGGTGCGGATCTGGCGGC<br>CTACAACGCTGCGCTAAGCTGCAACGAAAACGACGCAGAGCACCTGG<br>ACCGTGACCAGCCATACCCGAGCAGCCAGAAGACCAAGCGCATGCGC<br>ACGTCCTTCAAGCACCACCAGCTTCGGACCATGAAGTCTT
LTBP4	CGAGTACGGCCCAGACTTAGGTCCACCTTACCAGGGCCTCCCATATG<br>GGCCTGAGTTGTACCCACCACCTGCGCTACCCTACGACCCCTACCCA<br>CCGCCACCTGGGCCCTTCGCCCGCCGGGAGGCTCCTTATGGGGCAC<br>CCCGCTTCGACATGCCAGACTTTGAGGACGATGGTGGCCCCTATGGC<br>GAATCTGAGGCTCCTGCGCCACCTGGCCCGGGCACCCGCTGGCCCTA<br>TCGGTCCCGGGACACCCGCCGCTCCTTCCCAGAGCCCGAGGAGCCTC<br>CTGAAGGTGGAAGCTATGCTGG
ANXA3	GTTGGACACCGAGGAACAGTAAGAGATTATCCAGACTTTAGCCCATCA<br>GTGGATGCTGAAGCTATTCAGAAAGCAATCAGAGGAATTGGAACTGAT<br>GAGAAAATGCTCATCAGCATTCTGACTGAGAGGTCAAATGCACAGCGG<br>CAGCTGATTGTTAAGGAATATCAAGCAGCATATGGAAAGGAGCTGAAA<br>GATGACTTGAAGGGTGATCTCTCTGGCCACTTTGAGCATCTCATGGTG<br>GCCCTAGTGACTCCACCAGCAGTCTTTGATGCAAAGCAGCTAAAGAAA<br>TCCATGAAGGGCGCGGGAACAAACGAAGATGCCTTGATTGAAATCTTA<br>ACTACCAGGACAAGCAGGCAAATGAAGGATATCTCTCAAGCCTATTAT<br>ACAGTATACAAGAAGAGTCTTGGAGATGACATTAGTTCCGAAACATCTG<br>GTGACTTCCGG
SMAD7	GTGGCATACTGGGAGGAGAAGACGAGAGTGGGGAGGCTCTACTGTGT<br>CCAGGAGCCCTCTCTGGATATCTTCTATGATCTACCTCAGGGGAATGG<br>CTTTTGCCTCGGACAGCTCAATTCGGACAACAAGAGTCAGCTGGTGCA<br>GAAGGTGCGGAGCAAAATCGGCTGCGGCATCCAGCTGACGCGGGAG<br>GTGGATGGTGTGTGGGTGTACAACCGCAGCAGTTACCCCATCTTCATC<br>AAGTCCGCCACACTGGACAACCCGGACTCCAGGACGCTGTTGGTACA<br>CAAGGTGTTCCCCGGTTTCTCCATCAAGGCTTTCGACTACGAGAAGGC

	GTACAGCCTGCAGCGGCCCAATGACCACGAGTTTATGCAGCAGCCGT<br>GGACGGGCTTTACCGTGCAGATCAGCTTTG
ANXA7	AGAACTGATCCTGGCCCTCTTCATGCCTCCTACGTATTACGATGCCTG<br>GAGCTTACGGAAAGCAATGCAGGGAGCAGGAACTCAGGAACGTGTAT<br>TGATTGAGATTTTGTGCACAAGAACAAATCAGGAAATCCGAGAAATTGT<br>CAGATGTTATCAGTCAGAATTTGGACGAGACCTTGAAAAGGACATTAG<br>GTCAGATACATCAGGACATTTTGAACGTTTACTTGTGTCCATGTGCCAG<br>GGAAATCGTGATGAGAACCAGAGTATAAACCACCAAATGGCTCAGGAA<br>GATGCTCAGCGTCTCTATCAAGCTGGTGAGGGGAGACTAGGGACCGA<br>TGAATCTTGCTTTAACATGATCCTTGCCACAAGAAGCTTTCCTCAGCTG<br>AGAGCTACCATGGAGG

Supplementary table 2. List of bisulfite primers used

Gene	Primer Forward	Primer Reverse	Amplicon size
OSTN	AGGGTTTTTATGGAAAAGAAATTTT	AATTACATATATCATAATTAACATTAATTC	211 bp
MYB	AAAGGGGTTTTTATTTTTGTAAATTGT	ATAAAAAAACTACACCCATCTTCCC	178 bp
LHX2	TTTTTTGAAAGAAAGTGTTTTAGATG	AAAAAAAACCACTAAACCCAAAAAC	156 bp
BMP6	TTTTTTTTATGTTGGATTTGTATAA	CCCAAATTCACAAAACTCATAAC	268 bp
SMAD5	GTTTAGATTTTGGAGGGAGG	ATTCATCAAACCATTAAACAACTTC	192 bp
LTBP4	TTAGAATTGGGAGTAGAGAGTTGAG	TCAAAAAACAACAAAAACAAATACC	232 bp
ANXA3	GAGTTGGAGGGATTTTAGTTTTGTAT	TTTTTCTATCCCCTAAACCTTTAAAAC	222 bp
PAX7	GGAGTTTTTGGGATTTATATTTGTT	AAATCCAAATCTACCAAAATTTCAAC	124 bp
SMAD7	TATTGGTTTTGTTTTAAAGGTTTTG	CACACTTCTCCAAACAATCCTAAA	250 bp
ANXA7	TATTTGAGGGAAAAAGATTTTTTTT	AAACTACACCAACCTCCCAATC	198 bp
