# Supplementary material for: MicroRNAs promote skeletal muscle differentiation of mesodermal iPSC-derived progenitors
Source: Nat Commun. 2017 Nov 1;8:1249. doi: 10.1038/s41467-017-01359-w (PMC5665910; doi:10.1038/s41467-017-01359-w)
Supplement: Supplementary file 3 — Description of Additional Supplementary Files [file 41467_2017_1359_MOESM3_ESM.pdf]

Description of Additional Supplementary Files

File Name: Supplementary Data 1
Description: List of GO terms of f-MiPs and MAB- MiPs.

File Name: Supplementary Data 2
Description: mRNAs and miRNAs differentially expressed between fMiPs and MAB-MiPs.

File Name: Supplementary Data 3
Description: List of differentially expressed genes between MiPs treated and untreated.
